# Prévalences du syndrome métabolique et des facteurs de risque cardiovasculaire chez les diabétiques de type 2 vus au service d’endocrinologie, Antananarivo

**DOI:** 10.11604/pamj.2020.36.67.15845

**Published:** 2020-06-04

**Authors:** Sitraka Angelo Raharinavalona, Thierry Razanamparany, Rija Eric Raherison, Andrinirina Dave Patrick Rakotomalala

**Affiliations:** 1 Service d’Endocrinologie, Hôpital Joseph Raseta de Befelatanana, Antananarivo, Madagascar,; 2 Service d’Endocrinologie et de Cardiologie, Centre Hospitalier Universitaire Mahavoky Atsimo, Mahajanga, Madagascar

**Keywords:** Antananarivo, diabète de type 2, facteurs de risque cardiovasculaire, syndrome métabolique, Antananarivo, type 2 diabetes, cardiovascular risk factors, metabolic syndrome

## Abstract

Le syndrome métabolique (SM), étant considéré comme un facteur de risque cardiovasculaire, constitue un problème majeur de santé publique. Il aggrave les risques déjà élevés chez les diabétiques. Notre étude vise à déterminer les prévalences du syndrome métabolique et des autres facteurs de risque cardiovasculaire (FDR CV) associés chez les diabétiques de type 2. Il s’agit d’une étude transversale descriptive et analytique, menée au sein du Service d’Endocrinologie du Centre Hospitalier Universitaire Befelatanana, Antananarivo, sur une période de 7 mois. Le diagnostic du SM était posé selon les critères du consensus d'harmonisation de l’*International Diabetes Federation* (2009). Nous avons retenu au total 219 patients diabétiques de type 2 dont 189 avaient présenté un SM donnant une prévalence de 86,30%. Leur âge moyen était de 58,58 ans avec une prédominance féminine (55,88%). Leur diabète évoluait, en moyenne, depuis 4,36 ans. A part l’hyperglycémie, l’hypertension artérielle était la composante du SM la plus observée, suivi de l’hypoHDLémie, de l’obésité abdominale et l’hypertriglycéridémie chez les deux genres. Les autres FDR CV associés au diabète le plus observé étaient la dyslipidémie, suivie de la surcharge pondérale ou l’obésité, de l’albuminurie et du tabagisme. Seul le surpoids ou l’obésité était le FDR CV corrélé significativement avec le SM. La prévalence du SM était très élevée chez nos diabétiques de type 2 qui cumulaient plusieurs autres FDR CV. Une prise en charge adéquate de ces différents FDR sera donc nécessaire pour réduire le SM et ses conséquences afin d’améliorer leur survie.

## Abstract (en)

Metabolic syndrome (MS) is considered as a cardiovascular risk factor and has become a major public health issue. It exacerbates the risks, which are already high among diabetics. The purpose of this study is to determine the rates of metabolic syndrome and other cardiovascular risk factors (CRFs) in type 2 diabetics. We conducted a cross-sectional descriptive and analytical study in the Department of Endocrinology at the Befelatanana General Hospital, Antananarivo, over a period of 7 months. The diagnosis of MS was based on IDF harmonized criteria (2009). A total of 219 patients with type 2 diabetes were involved in the study, of whom 189 had metabolic syndrome (86.30%). MS was predominant in female patients (55.88%). On average, the mean age of patients was 58,58 years, BMI was 24.28 kg/m2 and abdominal circumference was 87,40 cm. Diabetes developed, in average, over 4.36 years (75.8% of patients had imbalance). Apart from hyperglycemia, arterial hypertension (AH) was the most common component of MS, followed by hypoHDLemia, abdominal obesity and hypertriglyceridemia between the two genders. Other most commonly reported cardiovascular risk factors associated with diabetes were dyslipidemia, followed by overweight or obesity, albuminuria and smoking. Overweight or obesity were cardiovascular risk factors significantly associated with MS. MS rate was very high in type 2 diabetics with several other CRFs. Adequate management of these risk factors is necessary to reduce the number of patients with MS as well as its consequences in order to improve survival.

## Introduction

Le syndrome métabolique (SM) correspond à la coexistence de plusieurs désordres métaboliques dont trois facteurs parmi cinq chez un même individu. Ces cinq critères majeurs sont l’obésité centrale ou abdominale, hypertriglycéridémie, la baisse de High-Density Lipoprotein cholesterol (HDLc), l’hyperglycémie, et l’élévation de pression artérielle [1]. Il a fait l'objet de diverses définitions au cours des 10 dernières années. Il s’agit d’une entité clinico-biologique reconnue par l’Organisation Mondiale de la Santé (OMS) en 1998, le *National Cholesterol Education Program-Adult Treatment Panel III* (NCEP-ATP III) en 2001 [2], l’*International Diabetes Federation* (IDF) en 2005 [3], puis le consensus d'harmonisation de l’IDF en 2009 [4]. Sa prévalence dépend de l’âge, de l’origine ethnique de la population d’étude et surtout du critère de diagnostic retenu. Elle varie entre 17,9 à 80% au sein de la population diabétique [5, 6]. Les risques de maladie cardiovasculaire et de décès associé au SM sont respectivement multipliés par 1,7 et 1,3 [7, 8]. L'association d'autres facteurs comme le SM, augmente le risque vasculaire chez les patients diabétiques déjà à haut risque. En outre, le diabète constitue la première cause de mortalité cardiovasculaire [9]. Tout ceci traduit ainsi l'importance de ce problème. En l'absence de données sur l'ampleur du phénomène parmi les diabétiques dans notre pays, nous avons réalisé cette étude. Elle avait pour objectif de déterminer la prévalence du SM et des autres facteurs de risque cardiovasculaire (FDR CV) associés chez les diabétiques de type 2 afin de suggérer des mesures préventives et d’améliorer leur prise en charge.

## Méthodes

Nous avons réalisé une étude transversale descriptive, au sein de Service d’Endocrinologie du Centre Hospitalier Universitaire Joseph Raseta de Befelatanana (CHU-JRB), Antananarivo. L’étude a été menée sur une période de 7 mois (novembre 2016 au mai 2017). Pour être inclus dans l’étude, les patients ont dû être diabétiques de type 2 connus ou nouvellement diagnostiqués, hospitalisés dans le site d’étude, acceptant de participer à l'étude. Le diagnostic du diabète et leur typage étaient établis selon les critères de l’American Diabetes Association (ADA) 2010 [10].

Les patients présentant un état d’anasarque et/ou enceinte, une hypothyroïdie ou une tumeur maligne quelconque pouvant influencer les paramètres lipidiques ou n’ayant pas pu bénéficier les examens paracliniques requis, ont été exclus de notre étude. Les paramètres retenus étaient le genre et l’âge du patient, la durée d’évolution du diabète, la glycémie à jeun et le taux de l’Hb A1C, la présence du SM, le nombre de ses composantes et les autres FDR CV associés (dyslipidémie, surpoids ou obésité, albuminurie et tabagisme). Les patients ayant un ou plusieurs paramètres lipidiques en dehors des cibles recommandées par l’ADA 2010 ont été considérés comme ayant une dyslipidémie [10].

Le diagnostic du SM était posé selon les critères du consensus d'harmonisation de l’IDF en 2009 dont la présence de 3 au moins des 5 critères suivants [4]: une obésité abdominale: correspondant à un tour de taille supérieur ou égal à 94 cm chez l'homme et 80 cm chez la femme ; une triglycéridémie supérieure ou égale à 1,50 g/l (ou 1,7mmol/l) et/ou une prise d'un traitement hypolipémiant spécifique ; un taux de HDL-cholestérol inférieur ou égal à 0,40g/l (1,03 mmol/l) chez l'homme et 0,50 g/l (1,29 mmol/l) chez la femme et/ou une prise d'un traitement hypolipémiant spécifique ; une pression artérielle supérieure ou égale à 130/85 mmHg ou une hypertension artérielle (HTA) sous traitement ; une glycémie à jeun élevée, supérieure ou égale à 1 g/l (5,6 mmol/l) ou une prise d'un traitement antidiabétique.

Les données ont été recueillies à l’aide d’une fiche préétablie et exploitées par le Logiciel R avec un test significatif p value < 0,05. Tous les patients concernés par l’étude avaient consenti à y participer et avaient signé une fiche de consentement éclairé.

## Résultats

Durant la période d’étude, 663 diabétiques ont été hospitalisés dans le site. Nous avons retenu au total 219 patients diabétiques de type 2 ayant répondu aux critères d’éligibilité. Parmi ces patients retenus, 189 avaient présenté un SM donnant une prévalence de 86,30%.

**Figure 1:**
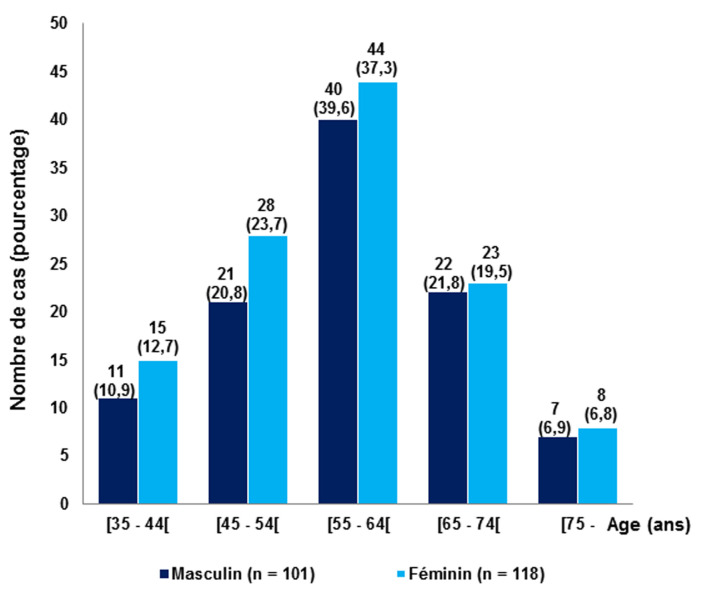
: répartition des patients selon la tranche d’âge

Le Tableau 1 présente les caractéristiques générales de notre population d’étude. Elle était composée de 101 hommes (46,12%) et 118 femmes (53,88%), donnant un sex-ratio de 0,86. Chez tous les genres confondus, l’âge moyen était de 58,58 ± 11,10 ans, avec des extrêmes de 35 à 88 ans. Les patients étaient âgés entre 55 et 64 ans dans 38,36% de cas et entre 45 et 54 ans dans 22,37% de cas (Figure 1). L’indice de masse corporelle (IMC) moyen était de 23,95 ± 3,55 kg/m^2^ chez le genre masculin et de 24,57 ± 4,74 kg/m^2^ chez le genre féminin. Dans 62,56% de cas, les patients avaient une corpulence normale. Le périmètre abdominal (PA) était, en moyenne, de 89,82 ± 10,95 cm chez les hommes et de 85,33 ± 13,72 cm chez les femmes. La durée moyenne d’évolution du diabète était de 4,36 ± 5,83 années, avec des extrêmes de 0 à 40 années. En moyenne, la glycémie à jeun était de 11,04 ± 6,42 mmol/L et l’hémoglobine glyquée était de 8,73 ± 2,51% dont la plus élevée était de 16,8%. Seuls 53 patients (24,2%) avaient un diabète équilibré avec une Hb A1C < 7%. L’âge, l’IMC, le PA, l’ancienneté du diabète, la glycémie à jeun et l’Hb A1C n’avaient pas influencé l’existence d’un SM.

**Tableau 1:** caractéristiques générales de la population d’étude

caractéristiques des patients	Total (N = 219)	Homme (n = 101)	Femme (n = 118)	p value
Age moyen (ans)	58,58 ± 11,10	58,87 ± 10,56	58,33 ± 11,59	0,284
IMC moyen (kg/m2)	24,28 ± 4,23	23,95 ± 3,55	24,57 ± 4,74	0,932
**Degré de corpulence (n) (%)**				
Maigreur et normale	137 (62,56)	63 (62,38)	74 (62,71)	---
Surcharge pondérale	64 (29,23)	34 (33,66)	30 (25,42)	---
Obésité modérée	14 (6,39)	3 (2,97)	11 (9,32)	---
Obésité sévére	2 (0,91)	1 (0,99)	1 (0,85)	---
Obésité morbide	2 (0,91)	0 (0)	2 (1,70)	---
PA moyenne (cm)	87,40 ± 12,69	89,82 ± 10,95	85,33 ± 13,72	0,992
Durée moyenne d’évolution du diabète (ans)	4,36 ± 5,83	4,84 ± 5,14	3,96 ± 6,35	0,734
GAJ moyenne (mmol/L)	11,04 ± 6,42	11,17 ± 7,11	10,93 ± 5,78	0,672
**Hb A1C**				
Moyenne (%)	8,73 ± 2,51	9,02 ± 2,56	8,47 ± 2,45	0,429
< 7% (n) (%)	53 (24,2)	17 (16,83)	36 (30,51)	---
≥ 7% (n) (%)	166 (75,8)	84 (83,17)	82 (69,49)	---

IMC: indice de masse corporelle, PA: périmètre abdominale, GAJ: glycémie à jeun, Hb A1C: hémoglobine glyquée

**Figure 2:**
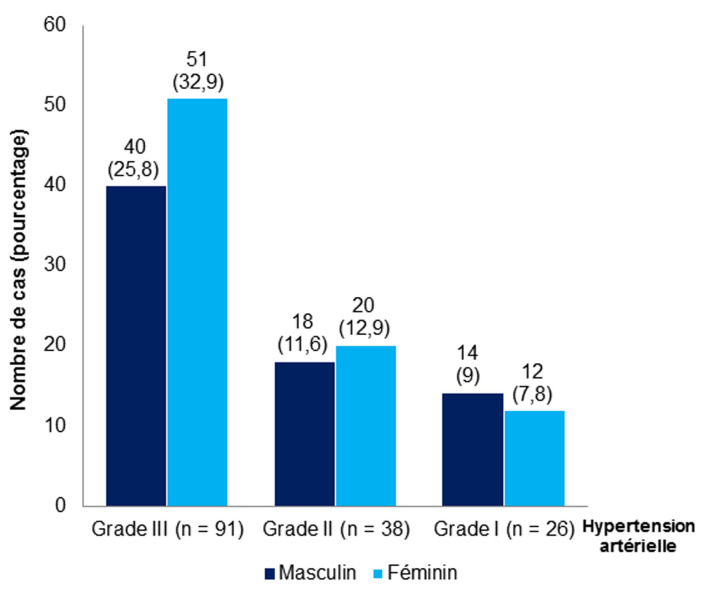
: répartition des patients selon le grade de l’hypertension artérielle

Concernant les composantes du SM, l’hyperglycémie était toujours présente. Cent cinquante-cinq patients (70,78%) avaient une HTA dont 58,71% étaient de grade III, 24,52% de grade II et 16,77% de grade I (Figure 2). L’HTA était associée significativement au SM (p < 0,001). L’hypoHDLémie (hypoHDL) était retrouvé dans 61,64% de cas, l’obésité abdominale dans 57,99% de cas et l’hypertriglycéridémie (hyperTG) dans 50,23% de cas avec p value respective à 0,015, < 0,001 et 0,011 (Tableau 2). Chez tous les genres confondus, le SM comportait 3 composantes dans 45,21% de cas, 4 composantes dans 28,31% de cas et 5 composantes dans 12,78% de cas (Tableau 3).

**Tableau 2:** répartition des patients selon les composantes du syndrome métabolique

Composantes du SM	Total (N = 219)	Homme (n = 101)	Femme (n = 118)	p value
Hyperglycémie n (%)	219 (100%)	101 (100%)	118 (100%)	---
Hypertension artérielle n (%)	155 (70,78%)	72 (71,29%)	83 (70,34%)	< 0,001*
HypoHDLémie cholestérol n (%)	135 (61,64%)	54 (53,47%)	81 (74,58%)	0,015*
Obésité abdominale n (%)	127 (57,99%)	50 (49,50%)	77 (65,25%)	< 0,001*
Hypertriglycéridémie n (%)	110 (50,23%)	52 (51,49%)	58 (49,15%)	0,011*

SM: syndrome métabolique, HDL: High-density lipoprotein. *p significatif < 0,05

**Tableau 3:** répartition des patients selon le nombre de composantes du syndrome métabolique

Nombre du composantes du SM	Total (N = 219)	Homme (n = 101)	Femme (n = 118)
2 ou 1 (n) (%)	30 (13,70)	15 (14,85)	15 (12,71)
3 (n) (%)	99 (45,21)	54 (53,47)	45 (38,14)
4 (n) (%)	62 (28,31)	23 (22,77)	39 (33,05)
5 (n) (%)	28 (12,78)	9 (8,91)	19 (16,10)

SM: syndrome métabolique

**Figure 3:**
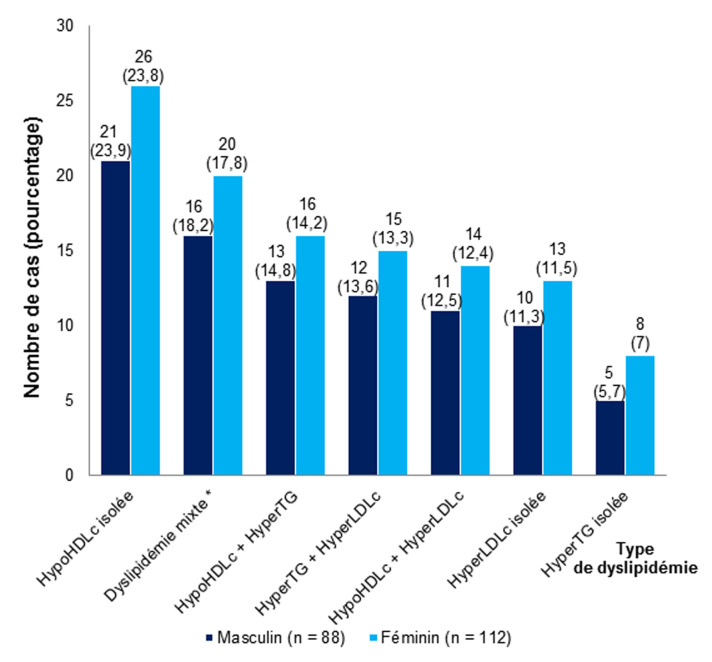
: répartition des patients selon le type de la dyslipidémie (HDLc: High density lipoprotein cholesterol, LDLc: Low-density lipoprotein cholesterol, TG: triglyceride, *Dyslipidémie mixte: association HypoHDL, HyperLDL et HyperTG)

La répartition des patients selon les autres FDR CV associés au diabète est résumée dans le Tableau 4. Tous nos patients avaient au moins un FDR CV associé à leur diabète. Dans 91,32% de cas, les patients étaient dyslipidémiques. L’hypoHDLémie isolée était le type de dyslipidémie le plus observé dans 23,5% de cas, suivi de la dyslipidémie mixte dans 18% de cas (Figure 3). Dans l’ensemble, la dyslipidémie n’avait pas influencé la présence du SM. Quatre-vingt-deux patients (37,44%) étaient en surcharge pondérale ou obèses. Le surpoids ou l’obésité était associé significativement à la présence du SM (p < 0,001). L’albuminurie était retrouvée chez 35,62% de patients mais sa présence n’était pas corrélée positivement avec celle du SM (p = 0,616). Cinquante-quatre (24,66%) patients étaient tabagiques actifs ou sevrés depuis moins de trois mois dont 31 de genre masculin et 23 de genre féminin.

**Tableau 4:** répartition des patients selon les autres facteurs de risque cardiovasculaire associés au diabète

Facteurs de risque cardiovasculaire*	Total (N = 219)	Homme (n = 101)	Femme (n = 118)	p value
Dyslipidémie (n) (%)				
Dyslipidémiques	200 (91,32)	88 (87,13)	112 (94,92)	0,719
Non dyslipidémiques	19 (8,68)	13 (12,87)	6 (5,08)	---
Surpoids ou obésité (n) (%)				
En surcharge pondérale ou obèse	82 (37,44)	38 (37,62)	44 (37,29)	< 0,001**
Maigre ou poids normal	137 (62,56)	63 (62,38)	74 (62,71)	---
Albuminurie (n) (%)				
Albuminuriques	78 (35,62)	43 (42,57)	35 (29,66)	0,616
Non albuminuriques	141 (64,38)	58 (57,43)	83 (70,34)	---
Tabac (n) (%)				
Tabagiques actifs ou sevrés moins de 3 mois	54 (24,66)	31 (30,69)	23 (19,49)	0,338
Non tabagiques	165 (75,34)	70 (69,31)	95 (80,51)	---

* : Tous nos patients avaient au moins un facteur de risque cardiovasculaire associé à leur diabète. **p significatif < 0,05

## Discussion

Le syndrome métabolique est de plus en plus considéré comme un problème majeur de santé publique [7]. Avec l’épidémie croissante de l’obésité et du diabète, la prévalence du SM rencontre aussi une augmentation exponentielle. Il est aussi reconnu comme étant un FDR CV majeur. Puisqu’il favorise la survenue d’athérosclérose et de dysfonction endothéliale. Il est aussi associé à un état prothrombique et à un état pro-inflammatoire [11]. Notre étude nous avait permis d’objectiver que la prévalence du SM était de 86,30%. Cette prévalence varie de 86% aux États-Unis [12], de 39,6 à 80% en Europe [6, 13], de 57,7 à 91,9% en Asie [14] et de 60,4 à 71,7% en Afrique Sub-Saharien [15, 16]. Ces différences pourraient s’expliquer par la diversité des populations étudiées que ce soit sur l'ethnie, le genre, l'âge et le poids corporel mais également par les définitions retenues permettant de diagnostiquer le SM. Néanmoins, la prévalence du SM reste particulièrement très élevée chez la population diabétique [12]. En effet, les personnes atteintes du SM sont trois à cinq fois plus susceptibles de développer un diabète de type 2 d’une part ; et l’hyperglycémie de l’état diabétique elle-même est l’une de composantes du SM d’autre part [12, 17]. Ce qui implique l’importance du dépistage systématique de ce syndrome chez les diabétiques de type 2.

Le sex-ratio de notre population d’étude était de 0,86. Ce qui rejoignait ceux des littératures où on notait également une prédominance féminine [18]. L’âge moyen de nos patients était comparable à celui de Tan *et al*. qui était de 55,7 ± 9,2 ans [19]. Dans notre série, l’âge n’était pas associé significativement avec le SM. Mais on constate que plus l’âge de nos patients avançait, plus ils présentaient un SM avec beaucoup plus de composantes. Certains auteurs ont affirmé que les personnes âgées sont plus susceptibles de développer un SM. Ce qui augmenterait le risque de développer plusieurs maladies chroniques chez ces sujets [20]. Chez les deux genres, nos diabétiques avaient un IMC moyen de 24,28 ± 4,23 kg/m^2^ dont quatre-vingt-deux (37,44%) étaient en surcharge pondérale ou obèse. Ce qui se rapprochait de celui d’une étude menée au Népal dont l’IMC moyen était de 26 kg/m2 [21]. En moyenne, le diabète de nos patients avait évolué de 4,36 ans dont 75,8% étaient déséquilibré. L’Hb A1C n’avait pas influencé l’existence d’un SM. Cependant, un diabète déséquilibré est associé à un risque accru d’un SM selon la littérature [22]. D’où la nécessité d’optimiser toujours l’équilibre glycémique de ces patients.

Puisque notre étude était faite sur une population diabétique, l’hyperglycémie était toujours présente. L’HTA était de grade III dans plus de la moitié de cas. Elle était la composante la plus observée, suivie de l’hypoHDL, de l’hyperTG et de l’obésité abdominale chez les hommes. Tandis que pour ces deux dernières composantes, l’obésité abdominale était plus représentative que l’hyperTG chez les femmes. Ce constat rejoignait celui d’une étude menée par Nsiah *et al*. au Ghana [23]. En effet, le tour de taille augmente avec une progression plus rapide chez les femmes que chez les hommes [24]. Dans notre étude, ces composantes étaient corrélées positivement au SM sauf l’hyperglycémie. Selon la littérature, ces composantes sont non seulement liées par des causes communes qui est le SM, mais également par l’effet commun à favoriser chacune des maladies cardiovasculaires [12]. En dehors du diabète et de l’HTA qui sont déjà des facteurs de risque cardiovasculaire majeurs, nos patients cumulaient d’autres facteurs. Plus de neuf patients sur dix présentaient une dyslipidémie dans notre série. Au cours du diabète de type 2, les anomalies lipidiques sont particulièrement fréquentes dont l’hypoHDLémie isolée qui est classique comme type de dyslipidémie selon plusieurs auteurs [25, 26].

L’obésité et la surcharge pondérale étaient retrouvées chez 37,44% de nos patients et elles étaient associées avec le SM. Ce résultat va dans le même sens qu’une étude réalisée à Ouagadougou [27]. Tout ceci pourrait s’expliquer par le fait que les personnes obèses sont souvent atteintes de diabète de type 2 et de dyslipidémie, par conséquent un SM. Plus d’un tiers de nos patients étaient albuminuriques et un quart tabagiques actifs ou sevrés depuis moins de trois mois. Ces FDR cardiovasculaire n’avaient pas influencés la présence SM dans notre étude. Cependant, leurs dépistage régulier et leur prise en charge, avec les autres FDR associés, sont toujours recommandés chez ces personnes atteintes du diabète de type 2 [10, 28]. Avec tous ces multiples FDR cumulés, nos patients étaient à haut risque cardiovasculaire méritant ainsi une prise en charge adéquate pour une meilleure prévention cardiovasculaire.

## Conclusion

La prévalence du SM était très élevée dans notre population d’étude, puisque plus de quatre diabétiques de type 2 sur cinq (86,30%) avaient présenté ce syndrome. De même, le diabète était associé dans 100% de cas avec au moins un autre FDR CV. Nos diabétiques cumulaient donc plusieurs FDR CV. Tout ceci constitue une bombe à retardement qui s’explosera tôt ou tard. Une prise en charge de ces différents FDR cardiovasculaire par l’éducation, un bon suivi du régime alimentaire, une activité physique adéquate et un éventuel traitement pharmacologique, sera donc nécessaire pour réduire de façon significative le SM chez les personnes atteintes du diabète de type 2 et par conséquence les risques de maladies cardiovasculaires y associés et ses complications.

### Etat des connaissances actuelles sur le sujet

- La prévalence du SM est particulièrement élevée chez les patients porteurs d’un diabète de type 2;
- Elle dépend surtout de l’origine ethnique de la population d’étude et du critère de diagnostic retenu;
- Ces patients porteurs de DT2 cumulent généralement d’autres facteurs de risques cardiovasculaires (FDR CV).

### Contribution de notre étude à la connaissance

- La prévalence du SM, chez les patients porteurs d’un DT2 à Antananarivo (Madagascar), était supérieure à celle des autres pays en Afrique Sub-Saharien; un dépistage systématique et une prise en charge adéquate de ce syndrome seraient alors primordiaux;
- Le DT2 était associé à d’autres FDR CV dans la majorité de cas; seuls, le surpoids ou l’obésité était corrélé significativement à la présence du SM;
- Néanmoins, une prise en charge globale des différents FDR CV modifiables serait toujours nécessaire pour réduire le SM chez les DT2 et ses conséquences.
